# Prospective Evaluation of Icu Neurostimulant Use for Acute Brain Injury (Prevail): A Pragmatic Observational Cohort Study

**DOI:** 10.21203/rs.3.rs-8744330/v1

**Published:** 2026-02-10

**Authors:** Richard Robert Riker, Haley R Torr, Sara Penrod, Stephanie C Chan, Angela Leclerc, Teresa L May, David B Seder, David J Gagnon

**Affiliations:** Maine Medical Center; Maine Medical Center; Maine Medical Center; Maine Medical Center; Maine Medical Center; Maine Medical Center; Maine Medical Center; Maine Medical Center

**Keywords:** acute ischemic stroke, intracerebral hemorrhage, central nervous system stimulant, amantadine, brain injuries, disorders of consciousness

## Abstract

**Introduction:**

Amantadine, an effective neurostimulant for traumatic brain injury, has limited data supporting its use for other acute brain injuries (ABI). This pragmatic study evaluated amantadine for nontraumatic ABI monitored in the ICU with the Coma Recovery Scale-Revised (CRS-R).

**Methods:**

This prospective open-label cohort study developed a protocol to treat impaired consciousness after ABI with amantadine, assessing participants with CRS-R as 0–100 Rasch units, monitoring feasibility, safety, and effectiveness.

**Results:**

40 patients (age 64 [52–73] years, 25 [62%] female) were included during this protocol development phase: 13 ischemic stroke, 10 intracerebral hemorrhage, 7 subarachnoid hemorrhage, 4 hypoxic-ischemic encephalopathy, 3 other ABI. The median baseline CRS-R was 11 (IQR 7–16), including 16 meeting criteria for Disorder of Consciousness. Among 30 participants with an adequate amantadine treatment trial, 27 (90%) responded, with disposition to home (1,4%), skilled nursing facility (6,22%), acute inpatient rehabilitation (15,56%), or withdrawal of life support (5,18%). Pre- versus post-treatment CRS-R increased for responders by 8 (6–11), equivalent to 26 (15–45) Rasch units. Among 10 untreated or inadequate trial patients, 3 (30%) were discharged to rehab, 7 (70%) had support withdrawn.

**Conclusions:**

It is feasible to measure responsiveness to amantadine using the CRS-R for ICU patients with ABI.

Clinical practice guidelines and roundtables recommend early rehabilitation after stroke and acute brain injury,^1,2,3^ emphasizing multimodal interventions (e.g., medications and therapy); early recovery and pharmacological interventions are priorities for research.^3,4^ A recent systematic review examined neurostimulant administration after stroke, suggesting favorable responses in 70% of studies administering amantadine.^5^ Many outcome measures were utilized in the 22 studies reviewed, suggesting that no single indication for neurostimulant use nor measure of response has been identified.^5^ Among 10 studies administering amantadine in that review, only one publication included 5 patients during their acute post-stroke care.^6^ We previously reported our experience with neurostimulant use in 87 acute stroke patients, with 55% of patients receiving amantadine showing early improvement.^7^ Among several limitations identified, the Glasgow Coma Scale proved challenging to assess response to therapy, prompting us and others to suggest other tools for future studies.^3,7^ Other challenges reported in caring for ICU patients with acute severe brain injuries include identifying Disorders of Consciousness (DoC), monitoring patients serially, identifying common confounders of consciousness assessment, and intervening acutely to improve consciousness.^3^

We performed this prospective, single-center, observational cohort study with an interprofessional team to address these concerns, developing an amantadine treatment protocol for impaired consciousness related to acute, nontraumatic brain injuries (Figure 1). The decision to initiate amantadine in this pragmatic trial was made by the bedside team with input from clinical pharmacology, physiatry, physical and occupational therapy, and speech-language pathology. Participants were assessed with the Coma Recovery Scale-Revised (CRS-R), a validated, reliable, and recommended tool performed by trained Speech-Language Pathologists (SLP) experienced in assessing DoC.^3,8^ We also monitored the feasibility, safety, and effectiveness of this protocol. The MaineHealth IRB determined this treatment protocol development to be exempt from review [2102955–1].

We included adult patients with impaired consciousness associated with an acute brain injury, primarily focusing on acute ischemic stroke (AIS) or non-traumatic intracranial hemorrhage (ICH). Patients with other acute brain injuries (e.g. subarachnoid hemorrhage [SAH], hypoxic-ischemic encephalopathy [HIE] after cardiac arrest, or other causes) selected to receive amantadine were also monitored. Efforts to improve the clinical status of the patient by identifying and treating reversible causes prior to initiating amantadine therapy were also monitored.^3,7^

Based on potential confounders identified in prior studies,^3,5,7^ we excluded patients with a premorbid modified Rankin Scale (mRS) score ≥2, elevated intracranial pressure requiring pharmacologic intervention, those receiving amantadine for other indications (e.g., Parkinson’s disease), patients requiring continuous or scheduled sedation, those with active seizures requiring continuous electroencephalographic monitoring, and those with agitation or hyperactive delirium being treated pharmacologically. To provide comparative data during this pragmatic study, those assessed with the protocol but not treated with amantadine or those treated with amantadine for less than 96 hours were included a *priori* as a comparison group. We aimed to document the CRS-R prior to the first dose of amantadine and every 4–7 days.

Descriptive clinical outcomes included maximum amantadine dose, change in CRS-R from pre-treatment scores, and discharge disposition (acute rehabilitation, home, skilled nursing facility, hospice, or death). Severity of acute brain injury was documented with the National Institutes of Health Stroke Scale (NIHSS) for AIS,^9^ the ICH score for non-traumatic ICH,^10^ the Hunt-Hess score for SAH,^11^ the suppression ratio 6-hours after recovery of spontaneous circulation (SR6) for those with HIE after cardiac arrest,^12,13^ and pre-treatment admission Glasgow Coma Scale (GCS) for other etiologies of acute brain injury. Amantadine was administered with a starting dose of 100 mg administered twice daily at 06:00 and 14:00 with dose adjustments for renal dysfunction.

The primary effectiveness outcome of this pragmatic study was the consensus determination by the bedside clinical and rehabilitation team whether the patient was more responsive after initiating amantadine. We also monitored the CRS-R change from baseline to highest value prior to hospital discharge using the Rasch-transformed 0–100 measures of the CRS-R scores,^14,15^ with a threshold of 9 units.^15^ Safety parameters included need for new or increased sleep medication, new or worse agitation by Sedation-Agitation Scale scores,^16,17^ new myoclonus or seizure, hyperactive delirium requiring new pharmacologic treatment, and amantadine discontinuation or dose adjustment due to a suspected adverse effect. Continuous and ordinal variables are presented as median (interquartile range - IQR), nominal variables as number (%). No inferential statistical comparisons were planned in this observational study.

Forty patients were managed with this evolving protocol from January 2021 to February 2024, including 13 with AIS, 10 with ICH, 7 with SAH, 4 with HIE, 3 with infectious encephalitis, and 1 each with brain tumor, toxic ingestion, and severe sepsis encephalopathy. The median age was 64 (52–73) years, and 25 (62%) were female (Table 1). Sixteen (40%) participants met criteria for acute DoC (coma in 1, unresponsive wakefulness syndrome in 6, lower level minimally conscious state in 7, higher level minimally conscious state in 2), and 18 had milder cognitive-linguistic disorders.

Amantadine treatment was initiated in 32 participants a median of 9 (6–16) days after admission, but stopped after 4 or less days in two who were considered inadequately treated to assess for responsiveness; 8 patients never received amantadine per bedside team decision. Among the 30 participants with an adequate amantadine treatment course, 27 (90%) were considered responders who were discharged home (1;4%), to skilled nursing (6;22%), rehabilitation (15;56%), or transition to withdrawal of life support (WLS) (5;18%).

Six early participants were not assessed at baseline prior to initiating amantadine. The median baseline CRS-R was 11 (7–16) and 20 responders with pre- and post-CRS-R data had a median increase of 8 (6–11), equivalent to 26 (15–45) Rasch units (**Supplemental Table 1**). Serial CRS-R testing among 4 participants with delayed or no amantadine therapy and 4 with amantadine therapy are shown in Figure 2 as examples. All 20 responders with pre- and post-CRS-R data increased by 9 or more Rasch units, the minimal detectable change previously identified to exceed measurement error.^15^ The disposition for the 8 patients not treated with amantadine included 3 (38%) discharged to rehabilitation and 5 (62%) transitioned to WLS; both patients who received inadequate trials of amantadine transitioned to WLS. Among 6 participants with an initial CRS-R arousal subscale of 2, both treated patients were transferred to acute inpatient rehabilitation, while 2 of 4 untreated participants transitioned to WLS.

Among 32 participants receiving amantadine, 23 (72%) experienced no adverse drug effects, and 9 experienced one or more adverse drug effect potentially related to amantadine, including agitation in three which improved after amantadine was stopped or dose-reduced, and sleeplessness in three which improved despite continuation in one and after stopping in two. Amantadine was reduced from 200 mg to 150 mg twice daily in one patient due to intermittent myoclonus which improved with the dose reduction but was associated with a reduced CRS-R (Line 6, Figure 2). Seizures were suspected in two patients: amantadine was continued until WLS in one without further events and stopped after 5 days in another without further seizure activity. Several striking and rapid improvements in arousal were observed, including one patient untreated for a week with no improvement prompting a PEG to be scheduled, but then cancelled when wakefulness and swallowing improved dramatically within 48 hours of starting amantadine (Line 39 in Figure 2). Representative quotes from patient assessments are included in **Supplemental Table 2**.

This open-label, observational study showed that it is feasible to assess ICU patients with the CRS-R, evaluate potential confounders, and suggested that most participants receiving an adequate trial of amantadine for nontraumatic brain injury responded clinically and when comparing pre- and post-treatment assessments with the 0–100 Rasch transformation of the CRS-R.^14,15^ The CRS-R has been shown to be more reliable than the Glasgow Coma Scale,^3,18^ and SLP professionals were better able to obtain these data after identifying methods to facilitate assessment and communication, including emails and pharmacist contact when amantadine was ordered, SLP contact after amantadine was discussed on rounds, and establishing a larger pool of SLPs trained to perform the CRS-R. These advances to detect DoC among ICU patients, monitor them serially, and address potential confounders are needed for future successful clinical trials.^3^

As a non-randomized and unblinded study, potential bias and clinical improvement related to natural history rather than the intervention is possible, and these results must be considered hypothesis generating. We did not require that subjects met formal criteria for DoC to be treated in this cohort study, and many questions remain unanswered regarding use of amantadine during acute care of nontraumatic brain injuries, including disease- and anatomic location-specific responsiveness, severity of impairment most likely to respond, dose escalation and duration strategies, criteria for exclusion (e.g. prior restlessness or seizures, sympathetic storming, etc),^3,7^ and patient- and family-centered outcomes. These data and results may guide future neurostimulant trials in the ICU aiming to improve consciousness after brain injury.

## Supplementary Files

This is a list of supplementary files associated with this preprint. Click to download.

- 2026PREVAILSupplementalTable1.docx
- 2026PREVAILSupplementalTable2.docx

## Figures and Tables

**Figure 1:**
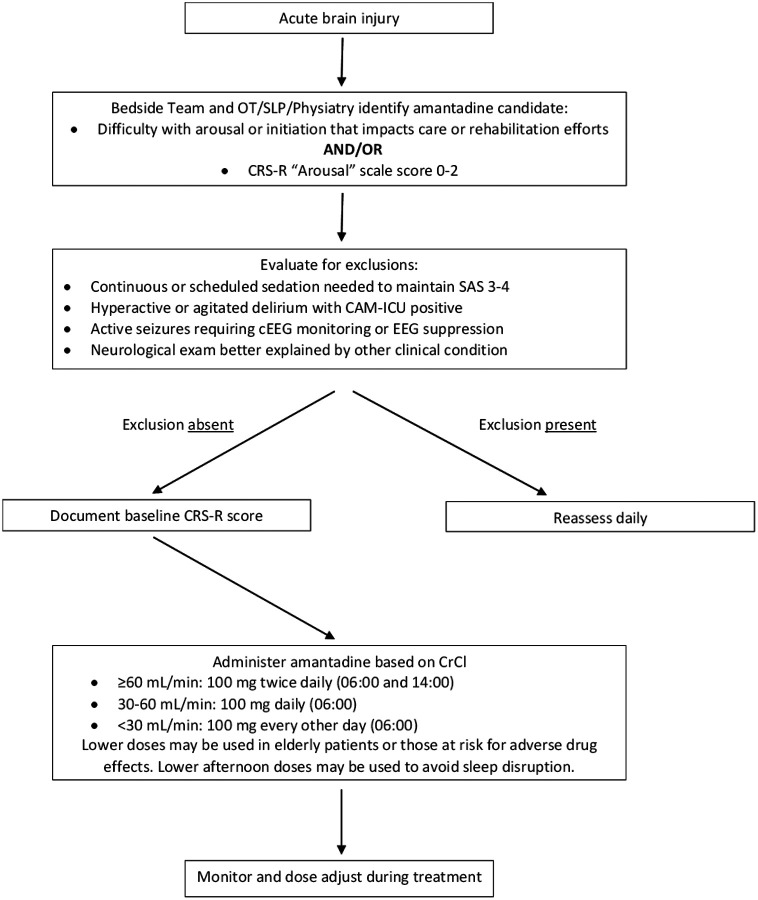
Amantadine Initiation Algorithm CAM-ICU, Confusion Assessment Method for the ICU; cEEG, continuous electroencephalography; CrCl, Creatinine Clearance; CRS-R, Coma Recovery Scale-Revised; OT, Occupational Therapy; SLP, Speech-Language Pathology; SAS, Sedation-Agitation Scale;

**Figure 2:**
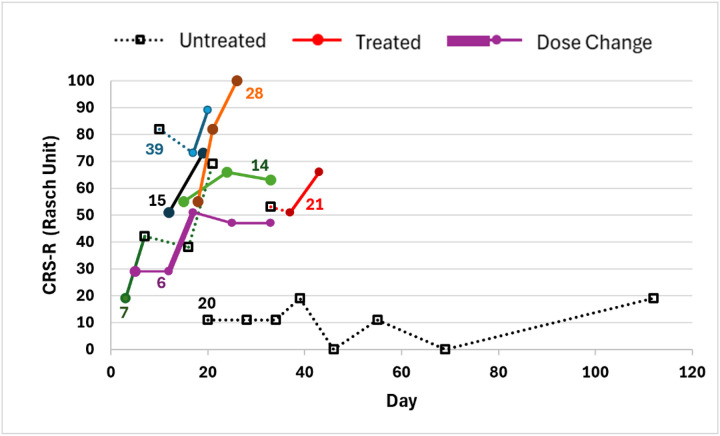
CRS-R Rasch Unit Over Time for 4 Treated and 4 Untreated or Delayed Treatment Participants Numbers identify participant from Table 1. Dotted lines and open squares represent no treatment periods. Solid circles and lines represent treatment periods. Fatter or skinnier lines represent an increase or decrease in amantadine dose (Line 6). Day represents day after hospital admission.

**Table 1:** Diagnoses, Demographics, Amantadine Dose and Response, Discharge Disposition

#	Diagnosis	Severity	Age	Gender	DOC	Respond	Start Day*--Max Dose	Rx@DC	Comment	Dispo
1	West Nile	GCS 11	72	M	--	Yes	22 -- 100 mg BID	No	Weaned off (sleep)	Rehab
2	ICH	ICH 3	35	F	MCS+	Yes	11 -- 200 mg BID	Yes	Emerged DOC	Rehab
3	ICH	ICH 4	28	F	MCS−	Yes	9 -- 200 mg BID	No	Family request to stop	SNF
4	ICH	ICH 2	46	M	--	Yes	3 -- 150 mg BID	Yes	See Quote	Rehab
5	SAH	HH 4	61	F	MCS−	Yes	9 -- 200 mg BID		Stopped at WLS	died WLS
6	CA HIE	SR6 50	65	M	UWS	Yes	6 -- 200 mg BID		Stopped at WLS	Hospice
7	IS	NIH 20	48	F	UWS	Yes	4 -- 150 mg BID	No	Stopped for seizure risk (no new events)	Rehab
8	IS	NIH 32	69	M	--	Yes	6 -- 150 mg BID	Yes	Modafinil added	Rehab
9	IS	NIH 26	60	M	MCS−	Yes	5 -- 100 mg BID	Yes	PEG discontinued	Rehab
10	ICH	ICH 0	61	M	--	No	8 -- 200 mg BID	--	Stopped at WLS	Hospice
11	IVH	ICH 5	68	M	CLD	Not Treated			See Quote	Hospice
12	SAH	ICH 4	64	M	CLD	Not Treated			See Quote	Rehab
13	SAH	HH 5	77	F	CLD	Yes	17 -- 100 mg BID	Yes	Improved – po diet	Rehab
14	SAH	HH 4	45	F	CLD/A	Yes	16 -- 100 mg BID	Yes	See Quote	SNF
15	ICH	ICH 3	74	M	CLD	Yes	14 -- 100 mg BID	--	Stopped at WLS	Hospice
16	IS	NIH 13	90	M	CLD/A	Short Treat	3 -- 100 mg BID	--	Stopped at WLS	Hospice
17	IS	NIH 38	48	F		Yes	21 -- 200 mg BID	Yes	Tracheostomy removed PEG discontinued	SNF
18	IS	NIH 18	78	F	CLD	Yes	9 -- 150 mg BID	Yes	PEG discontinued	Rehab
19	IS	NIH 22	64	M	CLD	Not Treated			See Quote	Rehab
20	IS	NIH 40	57	F	COMA	Not Treated			Not Rx due to PSH	died WLS
21	Brain Mass	GCS 9	79	M	MCS−	Yes	39 -- 200 mg BID	--	Continued until death	died IHCA
22	VPA Tox	GCS 4	54	F	--	No	23 -- 100 mg BID	No	Held after 5 days concern for seizures	Home Services
23	ICH	ICH 3	73	F	MCS−	Yes	6 -- 100 mg BID	Yes	See Quote	Rehab
24	CA HIE	SR6 99	64	M	MCS−	Yes	8 -- 100 mg BID	--	Stopped at WLS	died WLS
25	IS	NIH 32	58	F	MCS+	Yes	27 -- 100 mg BID	No	Stopped due to agitation -- not improved after	Rehab
26	ICH	ICH 3	65	F	CLD	Yes	6 -- 100 mg BID	--	Stopped at WLS	Hospice
27	SAH	HH 2	29	F	CLD/A	Yes	8 -- 100 mg BID	Yes	See Quote	Rehab
28	CA HIE	SR6 70	31	M	CLD	Yes	15 -- 100 mg BID	Yes	Tolerated after ICP and PSH controlled	Rehab
29	ICH	ICH 3	59	F	CLD	Yes	9 -- 200 mg BID	No	Stopped day 22 – delirium, poor sleep	Rehab
30	IS	NIH 33	85	F	CLD	Not Treated			Recommended by SLP, never started	died WLS
31	IS	NIH 30	69	F	CLD/A	Not Treated			Transferred out ICU	Rehab
32	ICH	ICH 4	80	M	UWS	Short Treat	3 -- 50 mg BID	--	“Never wanted this care”	died WLS
33	ICH-IVH	ICH 3	81	F	CLD	Not Treated			“No escalation care”	died WLS
34	Powassan	GCS 5	76	F	CLD	Yes	23 -- 100 mg BID	No	Stopped on floor – NPO severe ileus	Rehab
35	Enceph	GCS 6	64	F	MCS−	Yes	7 -- 200 mg BID	Yes	See Quote	SNF
36	IS	NIH 34	73	F	UWS	Not Treated			Transitioned WLS Hospital Day 2	died WLS
37	CA HIE	SR6 70	38	F	UWS	Yes	8 -- 200 mg BID	No	Agitation improved when weaned off	Home Services
38	IS	NIH 28	44	F	UWS	Yes	3 -- 200 mg BID	Yes	See Quote	Rehab
39	SAH	HH 2	72	F	CLD	Yes	18 -- 125 mg BID	Yes	Improved with RX, PEG cancelled	SNF
40	EEE	GCS 11	66	F	CLD	Yes	14 -- 100 mg BID	No	Stopped at discharge per Neurology	SNF

* Start Day – number of days from first hospital admission to first dose of amantadine; Max Dose - maximum dose of amantadine

BID, twice daily; CA HIE, cardiac arrest hypoxic-ischemic encephalopathy; CLD, cognitive-linguistic disorder; CLD/A, cognitive linguistic disorder with aphasia; DOC; Disorder of Consciousness; Dispo, disposition; EEE, Eastern Equine Encephalitis; Enceph, encephalopathy; F, female; GCS, Glasgow coma scale; HH, Hunt Hess; Home SVC, home with services; ICH, intracranial hemorrhage; ICP, intracranial pressure; IHCA, in-hospital cardiac arrest; IS, ischemic stroke; IVH, intraventricular hemorrhage; M, male; MCS, minimally conscious state; mg, milligrams; NIH, NIH Stroke scale; NPO, nothing by mouth; PEG, percutaneous endoscopic gastrostomy tube; Powassan, Powassan virus encephalitis; PSH, paroxysmal sympathetic hyperactivity; Rehab, inpatient rehabilitation; Rx@DC, Treated at Discharge; SAH, subarachnoid hemorrhage; SLP, Speech-Language Pathologist; SNF, skilled nursing facility; SR6, suppression ratio 6 hours after recovery of spontaneous circulation; UWS, unresponsive wakefulness syndrome; VPA tox, valproic acid toxicity; West Nile, West Nile encephalitis; WLS, Withdrawal of Life Support.
